# SH3 Domain Tyrosine Phosphorylation – Sites, Role and Evolution

**DOI:** 10.1371/journal.pone.0036310

**Published:** 2012-05-15

**Authors:** Zuzana Tatárová, Jan Brábek, Daniel Rösel, Marian Novotný

**Affiliations:** Department of Cell Biology, Faculty of Science, Charles University in Prague, Prague, Czech Republic; Universität Erlangen-Nürnberg, Germany

## Abstract

**Background:**

SH3 domains are eukaryotic protein domains that participate in a plethora of cellular processes including signal transduction, proliferation, and cellular movement. Several studies indicate that tyrosine phosphorylation could play a significant role in the regulation of SH3 domains.

**Results:**

To explore the incidence of the tyrosine phosphorylation within SH3 domains we queried the PhosphoSite Plus database of phosphorylation sites. Over 100 tyrosine phosphorylations occurring on 20 different SH3 domain positions were identified. The tyrosine corresponding to c–Src Tyr-90 was by far the most frequently identified SH3 domain phosphorylation site. A comparison of sequences around this tyrosine led to delineation of a preferred sequence motif AL**Y**D(Y/F). This motif is present in about 15% of human SH3 domains and is structurally well conserved. We further observed that tyrosine phosphorylation is more abundant than serine or threonine phosphorylation within SH3 domains and other adaptor domains, such as SH2 or WW domains. Tyrosine phosphorylation could represent an important regulatory mechanism of adaptor domains.

**Conclusions:**

While tyrosine phosphorylation typically promotes signaling protein interactions via SH2 or PTB domains, its role in SH3 domains is the opposite - it blocks or prevents interactions. The regulatory function of tyrosine phosphorylation is most likely achieved by the phosphate moiety and its charge interfering with binding of polyproline helices of SH3 domain interacting partners.

## Introduction

The SH3 domain is one of the most well characterized protein interaction modules. SH3 domain–mediated signaling is involved in all basic cellular processes as well as in many pathological conditions, including malignant transformation (reviewed in [1]).

SH3–mediated signaling processes are mostly driven by the recognition of polyproline–II helices by SH3 domain structures [2]. The SH3 domain ligand- binding surface plays a key role in intramolecular and intermolecular interactions [3]. It contains three hydrophobic pockets, each containing a cluster of conserved amino acid residues. Mutational analysis of SH3 domains identified key residues necessary for interactions with ligands. For example, the essential residues for Src SH3 domain ligand binding are Y90, N135 and Y136 in the first pocket, Y92, W118 and P133 in the second pocket, and D99 and Y131 in the third pocket (numbering based on chicken c-Src) [4]; [5].

Protein phosphorylation is one of the most fundamental regulatory events in eukaryotic cells [6]. The importance of reversible tyrosine phosphorylation in the regulation of essential cellular functions is underscored by the fact that tyrosine kinases comprise the largest group of oncoproteins [7].

Tyrosine phosphorylation is a relatively recent evolutionary innovation, having emerged approximately 600 million years ago, just prior to emergence of the first multicellular organisms [8]. The full phosphotyrosine signaling system including “writer” (kinase), “eraser” (phosphate) and “reader” (SH2 domain) is present in choanoflagellate *Monosiga brevicollis* [9]; [10], but elements of the system already appear in other unicellular organisms, such as *Acanthamoeba* [11].

During the past two decades, tyrosine phosphorylation within SH3 domains of several signaling proteins was discovered [12]; [13]; [14]. In some cases, mutational analyses were performed to determine the functional importance of a particular phosphorylated tyrosine. Results of these studies brought substantial evidence for a significant role of phosphorylation on the well conserved tyrosines within SH3 domain hydrophobic pockets in regulating the binding capacity of the SH3 domain and intramolecular regulation of signaling proteins. This mechanism of regulation seems to be used in various cellular processes and we hypothesize that it could be universally applicable to regulate signal transduction pathways mediated by proteins containing SH3 domains.

We surveyed available data from phosphoproteomic and structural studies to explore the abundance and variability of SH3 domain tyrosine phosphorylation sites, and to identify SH3 domain phosphorylation motifs. We also analyzed structural conservation of the AL**Y**D(Y/F) motif – the most frequently phosphorylated SH3 domain motif. Our results further support recent experimental observations that tyrosine phosphorylation within SH3 domains plays a critical role in the regulation of their function.

## Results and Discussion

### Survey of SH3 Domain Phosphorylation

SH3 domains are common protein interaction modules. Over 16000 SH3 domains in more than 12500 different proteins are described in the SMART database (October 2011). More than 97% of those occur in eukaryotic proteins. A growing body of experimental evidence indicates that tyrosine phosphorylation plays a significant role in regulation of many SH3 domains (Table 1).

**Table 1:** Summary of effects caused by mutation or phosphorylation at tyrosine sites in SH3 domains.

Protein	non-phospho-rylatable mutation	phospho-mimicking mutation	phospho-tyrosine	corresponding position in the alignment	Effect of mutation/phosphorylation	Ref.
Abi-1			Y398p	Y7	reduces binding to Abl	[33]
Abl	Y89F			Y7	decreases Bcr-Abl-mediated transformation ofTF-1 myeloid cells to cytokine independence	[13]
			Y89p	Y7	decreases interaction of SH3 domain withbinding partners both in *cis* and in *trans*	[16]
Btk	Y223F			Y7	blocks Btk autophosphorylation and potentiates the transforming activity of Btk in fibroblasts	[12]
			Y223p	Y7	disrupts the interaction with WASP	[34]
Crk			Y251p	Y17	induces Abl kinase transactivation	[35]
Grb2			Y209p	Y71	reduces binding to Sos	[36]
Itk	Y180F			Y7	plays positive role in Itk signaling	[37]
p130CAS	Y12F			Y7	decreases invasiveness in Src-transformed cells	[14]
		Y12E		Y7	decreases interaction of SH3 domain with FAK and PTP-PEST	[14]
			Y12p	Y7	decreases interaction of SH3 domain with FAK	[14]
PST-PIP		Y367E		Y7	decreases interaction with WASP	[38]
Endophilin		Y315E		Y7	decreases interaction of SH3 domain with Dynamin	[39]
ADAP			Y559p	Y66	positively affects interaction with Nck protein	[40]
CAP	Y623F			Y7	results in partial nuclear localization of CAP protein	[41]
Src	Y90A, Y92A			Y7, Y9	disrupts the interaction with Sam68 and PI3K-p85α	[4]
	Y133F,Y138F			Y66,Y71	inhibit PDGF and EGF mitogenic signaling	[42]
Txk			Y91p	Y7	contributes to upregulated IFN-g gene transcription	[43]
Vav1	Y826F			Y55	reduces binding to CSK	[44]

Tyrosine phosphorylation of SH3 domains has an unorthodox effect on protein function. Tyrosine phosphorylation is perhaps best known for its role in facilitating protein–protein interactions through the recognition of phosphotyrosine by a protein with a SH2 or PTB domain [15]. This usually leads to signal propagation. In contrast, the tyrosine phosphorylation of SH3 domains prevents or reduces the affinity of protein–protein interactions (Table 1). This can cause a switch in cell behavior, as in the case of chronic myeloid leukemia cells where phosphorylation of the SH3 domain of c–Abl enhances transformation potential [16], [13].

We queried the PhosphoSite Plus database for all phosphorylations within SH3 domains. At the time of the survey (October 2011), 188 distinct phosphorylation sites in 127 different SH3 domains were described in the database (File S1). Of these, 106 were tyrosine phosphorylations which were further analyzed.

**Figure 1:**
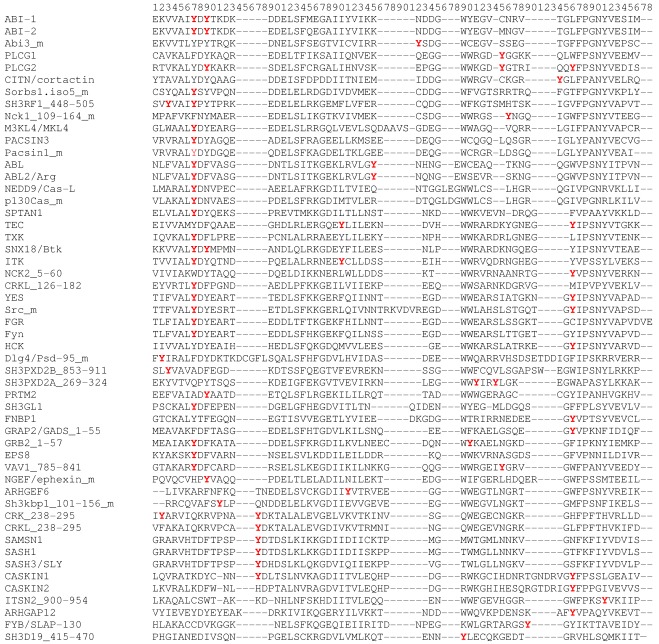
Multiple sequence alignment of tyrosine-phosphorylated SH3 domains. Phosphosite Plus database was searched for tyrosine phosphorylation within SH3 domains. The identified SH3 domains were aligned using ClustalW. Human sequences are shown except those depicted with **_m**, which come form mouse. All the sequences were obtained using SMART server. Names of proteins according to UniProt database are situated on the left including the domain range if there are more then one SH3 domains within a protein. Alignment is numbered at the top. Phosphorylated tyrosines are highlighted in red. Orthologous and paralogous sequences with identical phosphosites are not included.

SH3 domain sequences were aligned to determine the abundance of tyrosine phosphorylations at individual positions within the domain (Figure 1). To avoid redundancy, we included only one of the orthologue and paralogue (isoforms) sequences with identical phosphorylation pattern in the alignment. Fifty–two unique SMART–based SH3 domain sequences were aligned (File S2). A total of 36 protein domains were phosphorylated at one tyrosine site, 15 of them at two tyrosine sites and one (PLCγ2) on three sites.

We also analyzed the conservation of phosphorylation amongst orthologue sequences. We found 20 phosphorylated tyrosines that occurred in SH3 domains of two ortologues (Table S1) and eight phosphorylated tyrosines that were present in SH3 domains of three orthologues (Table S2). Our previous experimental data have further confirmed the phosphorylation of Tyr 12 in human, rat and mouse p130Cas [14]. Although available phosphorylation data are incomplete, we can conclude that a significant proportion of phosphosites (64 out of 106 in our survey) is present in more than one organism, further supporting the importance of SH3 domain tyrosine phosphorylation.

To unify the numbering of positions, we used the protein amino acid positions in the alignment in Figure 1 as our reference. The alignment showed that most tyrosine phosphorylations were detected at positions 7 and 66 (Table 2). These positions correspond to Y90 and Y131 in chicken Src SH3 domain localized, respectively, in the first and the third surface hydrophobic pockets. Therefore, both of these tyrosines are involved in ligand binding [17].

**Table 2:** Position-based phosphotyrosine abundance within SH3 domain.

**Phospho-Y position** **within SH3**	2	3	**7**	9	11	17	30	31	35	42	49	50	51	54	55	56	59	64	**66**	71
**Number of phoshorylations detected per site**	2	2	**24**	6	1	6	2	1	2	1	1	1	1	1	3	1	1	1	**11**	1

Phosphotyrosine position within SH3 refers to the position in alignment in Figure 1.

### Analysis of Abundant Phosphorylation Sites

**Figure 2:**
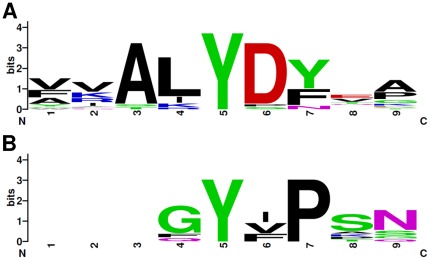
Weblogo of aligned segments of SH3 domains phosphorylated at Y7 and Y66 sites. Sequence logos were created using WebLogo (http://weblogo.berkeley.edu/logo.cgi) from 9aa long multiple sequence alignments (Fig. 1) with Y7 (A) and Y66 (B) in central position. The numbers of sequences for each WebLogo are indicated in upper left corner.

To further analyze the sequence surrounding the two most phosphor–enriched positions of Tyr 7 and 66, we created sequence logos (Figure 2, WebLogo [18]). The resulting consensus logos show an absence of strong amino acid conservation around Tyr 66 (with the exception of Pro on the position +2), while sequence around phosphorylated Tyr 7 is more conserved. Alanine at the position −2, leucine at position −1, and aspartate at the position +1 from Tyr 7, all show very strong conservation. The position +2 is predominantly occupied by amino acids with an aromatic ring – tyrosine and phenylalanine. Thus AL**Y**D(Y/F) is the most favorable motif for tyrosine phosphorylation in the SH3 domain. Since the sequence around Tyr 66 was not as well conserved and there were fewer observations of tyrosine phosphorylation on this site, further analysis was concentrated on the Tyr 7 site.

Of 304 human SH3 domains in the SMART database, the AL**Y**DY motif around Tyr 7 appears in 21 domains and the AL**Y**DF motif appears in 15 SH3 domains (File S3). Of those 36 sequences, 12 are known to be phosphorylated at Tyr 7 according to PhospositePlus. There are many domain definition programs available and they differ significantly in a number of predicted proteins with a particular domain. We therefore also evaluated the number of human SH3 domains with AL**Y**D(Y/F) sequence with an independent domain definition program – Pfam [19]. There are 750 human SH3_1 domains in the Pfam database. Among those, there are 113 sequences with either AL**Y**DY (64) or AL**Y**DF (49) motif (File S4 and S5). Results from the two independent domain definition systems roughly agree on the estimate that 12–15% of human SH3 domains possess AL**Y**D(Y/F) sequence motif that can potentially be phosphorylated.

Although it is very unlikely that all these motifs will get phosphorylated, we expect more experimental evidence on the significance of Tyr 7 phosphorylation in the near future due to an ever increasing amount of phosphosite data.

The high conservation of the sequence around Tyr 7 suggests that it could be phosphorylated by a specific group of kinases. We used GPS [20] and PhosphoMotifFinder [21] database/software to predict kinases responsible for the phosphorylation of Tyr 7 within the AL**Y**D(Y/F) motif. Both programs indicated Src–family kinases as the likely kinases for this site. GPS further suggested FAK, Btk, PDGF and Abl as potential kinases. AL**Y**DY motif of Itk was shown to by autophosphorylated by Itk itself [22]. This suggests that the phosphorylation of Tyr 7 is not mediated by a particular kinase in a specific cellular compartment. Rather, different kinase families working in different compartments of the cell may regulate SH3 domains through phosphorylation of the AL**Y**D(Y/F) motif.

### Structural Conservation of the ALYD(Y/F) Motif

Structural alignment was employed to further evaluate the AL**Y**D(Y/F) motif. There are 104 known 3D structures of SH3 domains with an AL**Y**D(Y/F) motif, representing 16 different proteins. A representative structure was selected for each protein and structurally aligned to c–Src structure (1FMK). Results of the structural alignment are shown in Table 2. The AL**Y**D(Y/F) motif is a part of the loop that connects the first and second strands in the structure. The loop folds in the conformation that is similar to the structure of two interacting strands in β–sheet (Figure S1). The conformation of the loop is (in the case of 1FMK) stabilized by three hydrogen bonds in–between the main chain atoms of amino acids within this loop: two hydrogen bonds between Tyr 9 and Phe 24 and by a hydrogen bond between Ala 5 and Gly 27. It is also stabilized by a hydrogen bond between the main chain atoms of Leu 6 and Tyr 71 that lies in the loop connecting strands four and five (numbering based on alignment in Figure 1).

**Figure 3:**
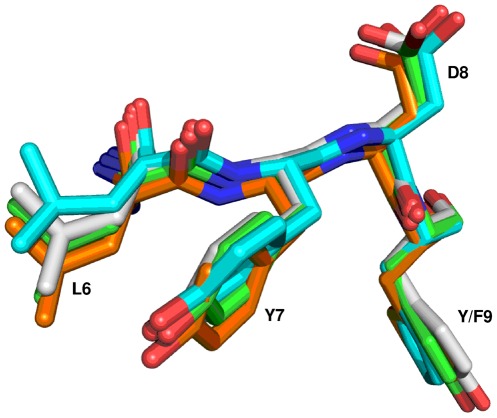
Structural alignment of ALYD(Y/F) motifs. Structures of human Src (green), human Abl (orange), yeast Pex13 (cyan) and Acanthamoeba Myosin Ib (grey) SH3 domains were aligned using LSQMAN. The whole SH3 domains were aligned. For the sake of clarity only the AL**Y**D(Y/F) motif is shown. The first amino acid of the motif (Ala) is hidden behind the plane of the figure. The phosphorylation on Tyr 7 (Y7) in the AL**Y**D(Y/F) motif was documented in mouse Src [45] and in human Abl [13]. The figure was created using PyMol.

The AL**Y**D(Y/F) motif is structurally well conserved. The root mean square distance (RMSD) for the C–alpha atoms in the motif was found to be typically less than half of the average RMSD for the whole SH3 domain (Table 3). Figure 3 further shows that even side chain conformations of the residues in this motif are very well conserved. The structural similarity holds true even for the AL**Y**D(Y/F) motif in proteins without experimentally verified phosphorylation in SH3 domains.

**Table 3:** Structural alignment of all SH3 domains with AL**Y**D(Y/F) motif with known 3D structure.

Structure	Protein	Organism	Motiv	Ex. Method	Seq. Identity	Average RMSD[Å]	RMSD (5–9)[Å]
1FMK	Src	human	AL**Y**DY	x-ray	100	0	0
2hda	Yes	human	AL**Y**DY	x-ray	76	1.02	0.39
3cqt	Fyn	chicken	AL**Y**DY	x-ray	71	1.04	0.22
4hck	Hck	human	AL**Y**DY	NMR	51	1.29	0.35
1aww	Btk	human	AL**Y**DY	NMR	42	1.45	0.81
1bbz	Abl	human	AL**Y**DF	x-ray	37	1.1	0.47
2d0n	Grb2 - related protein	mouse	AL**Y**DF	x-ray	33	1.04	0.4
1u06	Spectrin	chicken	AL**Y**DY	x-ray	33	1.1	0.37
2drm	Myosin	*A. castellanii*	AL**Y**DY	x-ray	32	1	0.18
2ed0	Abl2	human	AL**Y**DY	NMR	32	1.17	0.72
2v1r	Peroxin-13	*S. cerevisiae*	AL**Y**DF	x-ray	30	1.09	0.36
1x2q	Stam2	human	AL**Y**DF	NMR	30	1.19	0.38
3i5s	Pi3K	human	AL**Y**DY	x-ray	30	1	0.45
1yn8	Nap-1 binding protein	*S. cerevisiae*	AL**Y**DF	x-ray	28	1.07	0.46
1x69	Cortactin	human	AL**Y**DY	NMR	28	1.18	0.43
2yuq	Itk	human	AL**Y**DY	NMR	28	1.31	0.55

Structures were aligned to SH3 domain of human Src protein (1FMK). Two parameters are measured for each structural alignment – root mean square distance of the whole SH3 domains (Average RMSD) and of the AL**Y**D(Y/F) motif (RMSD (5–9)). Sequence identity to human 1FMK SH3 domain was calculated using ClustalW.

The RMSD values of the AL**Y**D(Y/F) motif did not correspond to sequence identity of aligned structures. Even the structures with a rather low sequence identity to c–Src had better RMSD values to c–Src than closely related proteins from Src kinase family. The best structural match of AL**Y**D(Y/F) motif of human c–Src was found in SH3 domain of myosin IB from *Acantamoeba castellanii* (2DRM, 32% seq. identity, 0.18 Å RMSD).

*Acanthameboa castellanii* belongs to Amebozoa, sister group to Opisthokonta (fungi and animals). Interestingly, according to a gene discovery study, *Acantamoeba castellanii* does contain basic elements of phosphotyrosine signaling pathway, including animal tyrosine kinase families, tyrosine phosphatases and proteins with SH2 domains [11].

A very good structural match was also found between AL**Y**D(Y/F) motif of human c–Src and those of two SH3 domains from *Saccharomyces cerevisie.* AL**Y**D(Y/F) motif occurs in four out of 29 *S. cerevisie* SH3 domains. However, animal tyrosine kinases have not been detected in yeast [15]. Nevertheless, this does not mean that phosphorylation on tyrosines does not occur in yeast. For example, kinase Swe1 inhibits the activity of Cdc28 by phosphorylation of its Tyr 19 [23].

The strong structural conservation of the AL**Y**D(Y/F) motif in Amebozoa and Opisthokonta could indicate that this mode of regulation is not a recent invention, but appeared before Amebozoa and Opisthokonta segregated.

### Tyrosine Phosphorylation Is Enriched in Other Docking Domains

We observed that tyrosine phosphorylations represent an unusually high proportion (68%) of all phosphorylations in SH3 domains,. We thus wanted to find out whether a prevalence of tyrosine phosphorylations is unique to SH3 domain or could be observed in other adaptor domains. We chose SH2, PH, PDZ, WW, PTB, EH, PX for further analysis [24]. Using PhosphositePlus database we searched for phosphorylation sites within these domains separately. We used only human proteins to avoid redundancy. For each domain we counted the ratio of tyrosine–phosphorylated sites to all of phosphorylations (Table 4). The statistics showed that there are 13324 (21,4%) human phosphotyrosine sites, 11618 (18,6%) human phosphothreonine sites and human 37410 (60%) phosphoserine sites in the PhosphoSitePlus database The tyrosine phosphorylation was overrepresented (in comparison to the database statistics) in five (out of seven) selected adaptor domains. There are three domains (SH2, WW and EH), where more than 50% of all documented phosphorylations are tyrosine phosphorylations. However, only a very few phosphorylations of EH have been observed. This suggests that tyrosine phosphorylation could also be an important regulatory mechanism for other adaptor domains. However, in human protein evolution tyrosine loss is strongly favored, most notably in protein subsets that are not known to be tyrosine phosphorylated (Tan-25). Thus the higher proportion of tyrosines in adaptor domains is in agreement with their higher tyrosine phosphorylation. Morover, the trend for enrichment of tyrosine phosphorylation in adaptor domains is maintained even after a correction to tyrosine content (Table 4).

**Table 4:** Ratios of phosphotyrosine to all phosphorylations within selected adaptor domains.

Domain	Number of pY to all phosphorylations	Ratio of pY to allphosphorylations [%]	Number of tyrosines (%)	Normalized relativepY enrichment
Human proteome	13324/62352	21,4	2,6	1,00
SH3	59/87	67,8%	3,8	2,17
SH2	72/104	69,2%	5,3	1,59
PH	75/159	47,2%	3,7	1,55
PDZ	13/73	17,8%	1,2	1,80
WW	11/14	78,6%	7,2	1,33
PTB	0/1	–	1,5	
EH	2/2	100%	2,0	6,08
PX	18/37	48,6%	3,7	1,60

The number of phosphorylations within selected domains was analyzed by PhosphoSite Plus and the ratio of phosphotyrosine sites (pY) to all sites was calculated. The complete human proteome from the Uniprot database was chosen to calculate the number of tyrosines among human proteins. The complete sets of human proteins containing adaptor domains was selected using the Pfam database.

Normalized relative enrichment of tyrosine phosphorylation shows ratio of percentage of pY to percentage of pY in human proteome normalized to number of tyrosines.

An other possible explanation of tyrosine phosphorylations enrichment in adaptor domains could be provided by the work of Fabian et al. which showed that while phosphorylation of serine residue had no impact on the structure of non–phosphorylated tau peptide, phosphorylation of the tyrosine results in considerable conformational changes [25].

In this study, we showed that tyrosine phosphorylation has been detected in a number of SH3 domains. The most phosphorylations have been detected at the position in the SH3 domain that is responsible for substrate binding. The experimental evidence shows that this tyrosine phosphorylation interferes with binding of SH3 domain to its interacting partners. We also showed that tyrosine phosphorylations occur frequently in other adaptor domains and could therefore represent an important regulatory mechanism of these domains.

## Materials and Methods

### Phosphorylation Search and Evaluation

All tyrosine phosphorylation sites in SH3 domains were identified in the PhosphoSite Plus database [26], curated and currently one of the most comprehensive databases of posttranslational modifications. For each hit from the PhosphoSite Plus, the occurrence of phosphorylation site in SH3 domain was carefully validated using the SMART (Simple Modular Architecture Research Tool) domain identification program [27]. Hits from PhosphoSite Plus that were not part of SMART–defined SH3 domains were not included in subsequent analyses. In case of doubt, the Uniprot annotation team was contacted for consultation, which led once to update of domain definition of a particular entry in the Uniprot database [28].

PhosphoSitePlus was also used to find tyrosine phosphorylation sites in other adaptor proteins. To avoid redundancy, we used only human proteins to calculate the ratio of tyrosine to all phosphorylations.

The occurrence of serines, threonines and tyrosines was calculated for the set of all human proteins as defined by Uniprot and compared to occurrence of these amino acids in the sets of all human SH3, SH2, PH, PDZ, PTB, EH,PX, WW domains as defined by Pfam [19].

Normalized relative phosphotyrosine enrichment was calculated as the ratio of tyrosine phosphorylations to the number of tyrosines in adaptor domains to the ratio of tyrosine phosphorylations to the number of tyrosines for all human proteins.

### Motif Definition and Motif Searches

The SH3 domains with identified tyrosine phosphorylations were aligned using ClustalW [29]. The alignment was further used to describe sequence motifs around two most frequently phosphorylated positions using WebLogo [18].

The AL**Y**D(Y/F) motif, identified around most frequently phosphorylated position 7, was used to estimate abundance of tyrosine phosphorylation in SH3 domains of human proteome.

Simple text search was used to locate AL**Y**D(Y/F) motif in all SH3 domains in the SMART and Pfam databases [27]; [19]. The Clustal W [29] was used to align sequences with identified AL**Y**D(Y/F) motif and sequences with AL**Y**D(Y/F) motif around Tyr 7 were selected.

GPS 2.1 (Group–based Prediction System) Online service [20] and PhosphoMotifFinder [21] were used to identify kinases that could phosphorylate tyrosines in ALYD(Y/F) motif.

### Structural Analysis

The PDB [30] was used to find all AL**Y**D(Y/F) motifs in SH3 domains with known 3D structures. One representative structure for each SH3 domain with more than one experimentally solved structure was selected. All selected 3D structures of SH3 domains with AL**Y**D(Y/F) motif were aligned to a reference SH3 structure (1FMK; [5], a high–resolution structure of human Src protein, using LSQMAN program [31]. The RMSD (Root mean square distance) for the whole SH3 domain and the described motifs were calculated and compared. ClustalW at the EBI webpage was used to calculate sequence identities between aligned structures [32]. PyMol was employed to visualize the results.

## Supporting Information

Figure S1 **β-sheet-like structure of a loop with ALYDY motif.** The AL**Y**DY motif is located in the loop that connects first and second β-strand in Src SH3 domain (1FMK). The loop conformation is stabilized by three hydrogen bonds in-between loop residues and by a hydrogen bond between Leu 6 and Tyr 71 (orange).
(TIF)

File S1 **UNIPROT codes of proteins with SH3 domain phosphorylation.**
(TXT)

File S2 **UNIPROT codes of proteins with tyrosine phosphorylation within SH3 domain.**
(TXT)

File S3 **UNIPROT codes of proteins with ALYDY/ALYDF motif within SH3 domain. Based on SMART database.**
(TXT)

File S4 **UNIPROT codes of proteins with ALYDY motif within SH3 domain. Based on Pfam.**
(TXT)

File S5 **UNIPROT codes of proteins with ALYDF motif within SH3 domain. Based on Pfam.**
(TXT)

Table S1 **List of phosphorylated tyrosines that occured in SH3 domains of two orthologues.**
(TXT)

Table S2 **List of phosphorylated tyrosines that occured in SH3 domains of three orthologues.**
(TXT)
