# Supplementary figures and images for: SH3 Domain Tyrosine Phosphorylation – Sites, Role and Evolution
Source: PLoS One. 2012 May 15;7(5):e36310. doi: 10.1371/journal.pone.0036310 (PMC3352900; doi:10.1371/journal.pone.0036310)

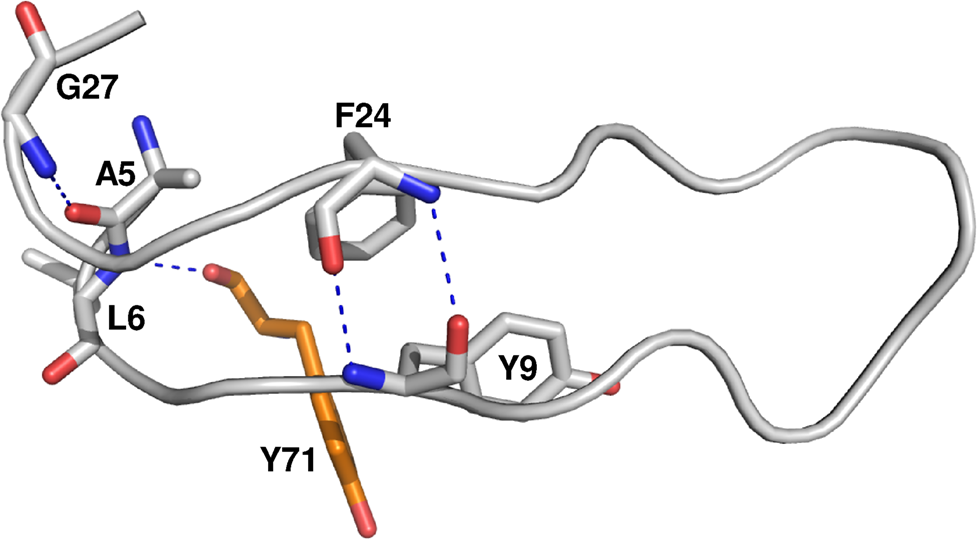

Supplement: Figure S1 — β-sheet-like structure of a loop with ALYDY motif. The ALYDY motif is located in the loop that connects first and second β-strand in Src SH3 domain (1FMK). The loop conformation is stabilized by three hydrogen bonds in-between loop residues and by a hydrogen bond between Leu 6 and Tyr 71 (orange). (TIF) [file pone.0036310.s001.tif]
